# The Cytoskeleton in Papillomavirus Infection

**DOI:** 10.3390/v3030260

**Published:** 2011-03-10

**Authors:** Malgorzata Bienkowska-Haba, Martin Sapp

**Affiliations:** 1 Department of Microbiology and Immunology, Louisiana State University Health Sciences Center, 1501 Kings Highway, Shreveport, LA 71130, USA; E-Mail: mbienk@lsuhsc.edu; 2 Feist Weiller Cancer Center, Louisiana State University Health Sciences Center, 1501 Kings Highway, Shreveport, LA 71130, USA

**Keywords:** papillomavirus, actin, microtubule, cytoskeleton, virus entry

## Abstract

Cytoskeleton defines the shape and structural organization of the cell. Its elements participate in cell motility, intracellular transport and chromosome movement during mitosis. Papillomaviruses (PV) are strictly epitheliotropic and induce self-limiting benign tumors of skin and mucosa, which may progress to malignancy. Like many other viruses, PV use the host cytoskeletal components for several steps during their life cycle. Prior to internalization, PV particles are transported along filopodia to the cell body. Following internalization, retrograde transport along microtubules via the dynein motor protein complex is observed. In addition, viral minichromosomes depend on the host cell machinery for partitioning of viral genomes during mitosis, which may be affected by oncoproteins E6 and E7 of high-risk human PV types. This mini-review summarizes recent advances in our understanding of papillomavirus’ interactions with the host cell cytoskeletal elements.

## 1. Introduction

Papillomaviruses (PV) replicate exclusively in the terminally differentiating epidermal cells of skin and mucosa in a strictly species-specific manner (for review see [1]). They infect the basal cells of this tissue [2], to which they probably gain access via microlesions. Following delivery of viral DNA to the nucleus of infected cells, infection is established by the initial amplification of the viral genome as nuclear minichromosomes. The infection spreads by cell division during which the viral genome copy number per cell remains constant. The viral life cycle is completed by amplification of the viral genome in fully differentiated cells, structural (late) gene expression, and viral assembly. Progeny virions are shed within the dead squames of the terminally differentiating keratinocytes. Human PV (HPV) comprises a large group of viruses with more than 100 genotypes identified so far. They usually induce benign self limiting tumors of skin and mucosa, which rarely progress to carcinomas of the cervix, vagina, penis, anus and others. Progression usually requires persistent infection by high risk types, like e.g., HPV16 and HPV18. Due to the high incidence of HPV in the general population, HPV infection is associated with more than 7% and 1% of cancers in women and men, respectively.

PV are small nonenveloped viruses with icosahedral symmetry. Their shell is composed of 360 molecules of the major capsid protein, L1, which are organized into 72 capsomeres, each comprised of a pentameric L1 assembly, forming a T = 7 icosahedral lattice [3]. In addition, up to 72 molecules of the minor capsid protein, L2, are incorporated into a virion supporting experimental evidence that L2 requires the pentameric L1 structure for interaction [4,5]. L2 is mainly hidden inside the capsid and only portions of the N-terminus (residues 60 to 120) are exposed on the capsid surface as determined by accessibility to antibody binding [6,7]. Additional indirect evidence suggests that the extreme N-terminus folds back into the capsid thus rendering it inaccessible to antibody binding and proteolytic cleavage in mature virions [8,9]. The capsid protects a circular double stranded DNA genome of approximately 8000 bp, which is organized into chromatin.

The strict dependence of PV on terminally differentiating keratinocytes for completion of their replication cycle initially made the study of entry processes difficult. First, it was impossible to propagate virions in cell culture, and viral yields from natural lesions were low, with some exceptions. The use of DNA-free virus-like particles (VLPs), virions generated in organotypic raft cultures and later pseudovirions harboring marker plasmids, which were generated using heterologous expression or cell-free packaging systems, helped to partially overcome this deficiency [10–17]. A second drawback hampering the study of PV entry, that is still not completely solved, is our inability to efficiently infect either organotypic raft cultures or primary keratinocytes *in vitro* [18] unless pseudovirions had been activated by furin precleavage of immature pseudovirions [19]. Therefore, researchers have had to rely on established cell lines, the most commonly used of which is the HaCaT cell line, to study PV binding and uptake. However, the recent development of an *in vivo* mouse model by the Schiller group will allow for the testing of observations made *in vitro*, despite the known deficiencies owing to switching species [2]. In this review we will focus on the role of the cytoskeleton in entry of high risk HPV type 16 (HPV16) and closely related viruses. Due to the experimental difficulties outlined above our knowledge is still rather limited. However, good progress has been made recently. *In vitro* data backed by *in vivo* studies suggest an elaborate sequence of cell surface events that includes retrograde flow along actin protrusions towards the cell body. In addition, it has become obvious that PV have evolved unique strategies for internalization and intracellular trafficking to overcome the challenges it faces by replicating in this terminally differentiating, stratified epithelium.

## 2. Summary of Current Knowledge

### 2.1. Receptors

In order to fully appreciate the role of cytoskeleton in PV entry, we have to briefly discuss primary and secondary receptors that are essential for initiating infectious internalization. The majority of PV types examined to date use heparan sulfate proteoglycans (HSPGs) as primary attachment receptors [20,21]. In HSPGs, unbranched oligosaccharides composed of alternating disaccharide units of uronic acid and glucosamine are linked to a protein core (syndecans, glypicans, perlecans) (reviewed in [22,23]). The carbohydrate chains are modified by sulfation to varying degree at the 2-*O*, 3-*O*, and 6-*O* position of the uronic acid and at the 3-*O* and 6-*O* position of the amino sugar. In addition, the amino group of the glucosamine may be either acetylated or sulfated. HSPGs can be cell surface associated (syndecans, glypicans) or secreted into the extracellular space (perlecans). *In vivo* studies using a cervicovaginal mouse model as well as *in vitro* studies suggest that genital HPV types, like HPV16, preferentially bind to the basement membrane and extracellular matrix (ECM), respectively. Even though binding to ECM-resident non-HSPG receptors has been demonstrated for some types *in vitro*, especially for HPV11, productive binding of HPV16 and HPV18 seems to require interaction with heparan sulfates [24–27]. Initial attachment is mediated by the major capsid protein L1 [28]. Some published evidence indicates distinct secondary interactions of L1 with HSPGs may occur subsequent to primary attachment [27,29]. Support for this came recently from the crystal structure of HPV16 and HPV18 L1 capsomeres in complex with heparin oligomers [30]. Four different modes of binding were seen with two binding sites each at the tip and the vertex of the capsomere. At least two L1 molecules within a capsomere contribute to a single binding site. Mutation of just one of these binding sites in HPV16 completely abolished both binding to ECM and cell surfaces [28,30], whereas exchanges in the other binding sites severely impaired infectivity without significantly reducing primary attachment [30]. This set of data suggests a secondary engagement of heparin binding sites. Not all HPV types may use HSPG as receptors. Raft-derived HPV31 virions were reported to not require HSPG interaction for infection of keratinocytes *in vitro* [31], however, HPV31 pseudovirions require HSPG in the cervicovaginal mouse model, similar to HPV16 [26]. Also, HPV5 infection was not sensitive to addition of heparan sulfates *in vitro* despite having detectable interaction [32]. However, it was blocked by heparan sulfates in the *in vivo* model, albeit with seemingly different requirements regarding sulfation [26].

### 2.2. Cell Surface Events

Two recent studies addressed the question of how the ECM/basement bound virions might be picked up by basal cells in order to gain cell entry. The Ozbun group reported that HPV31 virions induced a pronounced reorganization of the actin cytoskeleton after virus binding [33]. Cortical actin network and stress fiber break down was observed within 5 min of binding, followed by an induction of filopodia at 30 min post binding. Reorganization required signaling via tyrosine kinase and phosphoinositide 3 (PI3)-kinase. In addition, HPV31 infection was blocked by treatment with inhibitors of tyrosine and PI3 kinases. In the presence of inhibitors like genistein and wortmannin, virions were retained on the cell surface suggesting that signaling and cytoskeleton rearrangement is important for infectious entry. It was also shown that virions are taken up from ECM via retrograde transport along filopodia, filopodial retraction, and lateral curling of filopodia during which the filopodial tip was bent back towards the cell body. Indirect evidence that these transport processes occur on the cell surface came from neutralization studies following treatment with kinase inhibitors. More direct evidence for cell surfing of HPV16 virions along filopodia towards the cell body prior to internalization was provided by the Helenius group [34]. Here, electron microscopy and acid washes of FITC-labeled virions were used to demonstrate association of virions with the outer surface of filopodia. Fluorescence video microscopy was used to investigate virion transport along cell protrusions. The HPV16 virion transport speed matched the speed of F-actin retrograde flow. Actin flow requires three activities: polymerization of actin at the tip, anchored myosin II-mediated retrograde transport, and depolymerization at the cell body. Transport via actin was confirmed by the sensitivity of HPV16 directed flow to inhibitors of actin polymerization and depolymerization as well as to myosin II inhibitors. Additional observations suggested that retrograde transport was probably receptor-mediated. Virions showed directional movement at two different speeds, 1.6 and 2.9 μm/min, which indicates that transport occurs along two different kinds of actin protrusions. Directional movement was only observed along protrusions and not on the cell body. Furthermore, HPV16 pseudoinfection was impaired in scarce cell cultures treated with the myosin II inhibitor blebbistatin. Inhibition was not observed when dense cultures were infected, which have significantly lower numbers of actin protrusions. These findings indicate that retrograde transport on the cell surface contributes to efficient HPV16 infection. Indirectly, these data also suggest that virions are only internalized after they reach the cell body. It has become clear in recent years that a not yet identified secondary non-HSPG receptor is involved in infectious internalization of PV particles [8,27]. Which receptor links the virion to F-actin has not been addressed yet. It seems more likely that HSPGs play a role in this, as the handoff to the secondary receptor occurs rather late, directly before infectious internalization, and requires conformational changes affecting both capsid proteins. However, it was reported that conformational changes resulting in the exposure of the L2 N-terminus might occur at the basement membrane without requiring interaction with cell surfaces [35].

Attachment-induced conformational changes in L1 are not well defined yet but seem to involve the surface loop linking the antiparallel B and C β-strands [29,36], which are part of the capsomere core. Interaction with HSPG also induces a more clearly defined conformational change that results in the exposure of the L2 N-terminus [8]. This makes a highly conserved consensus furin convertase recognition site accessible for cleavage by furin convertase [37]. The cellular peptidyl-prolyl *cis*/*trans* isomerase Cyclophilin B (CyPB), which is associated with cell surface HSPG [38,39], facilitates the exposure of the L2 N-terminus [40]. Indirect evidence suggests that conformational changes reduce affinity of virions to HS, thus aiding the handover to the secondary receptor [8].

Evolution of such an elaborate process for gaining access to the basal cells may be warranted by the difficulties PV faces. First, PV infect the basal cells of the target tissue. These are believed to be the only cells within this tissue that support the establishment of PV infection. Second, access to these cells can only be efficiently achieved via microlesions. Thus, binding to receptors specific to the basement membrane prevents unproductive interactions with differentiated cells higher up in the tissue. In addition, virions will be preferentially taken up by basal cells migrating into the wound. During wound healing, these cells are triggered to allow for increased cell division, which again improves the likelihood of a successful infection.

### 2.3. Endocytosis

Probably owing to the complex events on the basement membrane and cell surface discussed above, internalization of PV is unusually slow and highly asynchronous. Internalization half times have been reported to range from 4 h for HPV16 to 14 h for HPV31 and HPV33 [20,41–43]. Evidence suggests that individual HPV types may use different endocytic pathways. Despite some contradictory reports, it has become clear in recent years that HPV16 is internalized via a clathrin-, caveolin-, and dynamin-independent novel pathway [44,45]. Entry and infection is resistant to individual and combined siRNA-mediated down regulation of caveolin-1 and clathrin heavy chain as well as to over-expression of dominant-negative mutants of dynamin-2, caveolin-1, and eps-15 (EGF receptor pathway substrate clone No. 15, which plays a role in clathrin coated vesicle formation) [45]. The use of a large library of siRNAs and inhibitors to interfere with known factors of endocytosis also excluded flotillin- and lipid raft-dependent endocytosis as well as macropinocytosis and phagocytosis as possible mechanisms of internalization [44]. Confirming early reports using VLPs [46], pseudovirions were found in wide uncoated pits up to 100 nm in diameter [44]. As of yet, this entry pathway has not been characterized any further but may utilize tetraspanin-enriched microdomains (TEM) as entry platforms. HPV16 pseudovirions accumulate at TEMs at the cell body, where they co-localize with keratinocytes-specific tetraspanin CD151. In addition, HPV16 infection is partially sensitive to treatment with CD151-specific antibody and siRNA [45]. This observation provides further support for the notion that internalization might be restricted to the cell body [34]. BPV1 was reported to utilize a clathrin-dependent endocytic pathway for infectious uptake based on a combination of microscopic analyses and biochemical inhibition of known pathways [47–49]. In contrast, HPV31 endocytosis seems to occur via caveolae [41,50]. However, one study found that treatment with chlorpromazine but not with inhibitors of caveolar uptake prevented HPV31 pseudovirus infection [51]. Additional, more systemic studies are required to address these discrepancies.

### 2.4. Cytosolic Transport

Passage through the cell membrane is followed by transport across the cytoplasm, uncoating, and nuclear entry of the genome. As outlined above, actin plays a critical role in endocytosis, not only being essential for cell surfing but probably also for cross-linking of receptor-ligand complexes as well as budding and release of endocytic vesicles from the plasma membrane [52]. Microtubules, in contrast, are involved in later steps including vesicular transport from early to late endosomes. Two early studies have analyzed the function of microtubules for these processes. Using HPV33 pseudovirions and various inhibitors, it was observed that actin is involved early in infection, whereas microtubules act at a later stage [53]. These results were essentially confirmed and extended using bovine PV type 1 (BPV1) virions and RT-PCR to assay infection [48]. Another study of BPV1 entry utilized immunofluorescence and electron microscopy. Liu *et al*. [54] observed virions in close association with microtubules immediately after infection of CV-1 cells. When the cells were kept at 37 °C, L1 protein accumulated in a perinuclear region. When the temperature was shifted from 37 °C to 4 °C or when cells were treated with nocodazole shortly after viral entry, leading to depolymerization of microtubules, BPV1 remained in numerous small vesicle-like structures scattered throughout the cytoplasm. However, a comprehensive study of vesicular intracellular trafficking of different PV types in normal keratinocytes is still lacking. Given the divergent reports regarding the endocytic mechanisms, it is not surprising that the subject of intracellular trafficking of PV-containing vesicles and the cellular compartments involved is also highly controversial [55].

There is near consensus that successful infection requires acidification of endocytic vesicles suggesting that PV particles must pass through the endosomal compartment [45,48,53,56]. It is believed that acidification is important for uncoating, which is required for the segregation of L1 protein from the L2/DNA complex. L1 protein accumulates in the lysosomal compartment, where it is probably degraded, whereas the L2/DNA complex escapes the endosomal compartment [57]. This requires functional L2 protein. Blockage of furin cleavage at the cell surface by mutation or inhibitors [37], inhibition of γ-secretase via an unknown mechanism [58,59], and mutations in a membrane-destabilizing domain of L2 [57] all prevent endosomal escape and abrogate infection.

Recent evidence suggests that microtubules play an important role in transport of viral genome not only during vesicular transport but also following egress from endosomes. The carboxyl-terminal 40 amino acids of HPV16 and HPV33 L2 were demonstrated to interact with the dynein motor protein complex, which mediates retrograde transport along microtubules. Mutational analysis suggested that this interaction is important for efficient infection [60]. Using yeast two-hybrid screens, co-immunoprecipitation, and immunofluorescence, Schneider *et al.* identified dynein subunits DYNLT1 and DYNLT3 as interaction partners of L2 [61]. The importance of these factors for HPV16 infection was confirmed by cytosolic delivery of DYNLT1- and DYNLT3-specific inhibitory antibodies and siRNA.

The issue of how the papillomaviral genome transits from the cytosol to the nucleus has not been systematically addressed. However, co-delivery of L2 and genome to the nucleus have been reported for HPV16 and BPV1, perhaps in conjunction with a cell-encoded chaperone [60]. Nuclear envelope breakdown is required for establishment of HPV16 infection indicating that active nuclear import via nuclear pore complexes may not be required [62]. However, it is unknown how the L2/DNA complex is retained in the reforming nuclei. It is undisputed that L2 protein accompanies the viral genome to the nucleus. L2 and the viral genome co-localize in the nucleus at PML nuclear bodies (PML NB; also called ND10 domains) following infection [63]. Efficient early PV transcription as well as transcription of the pseudoviral genome under the control of the CMV immediate early promoter requires either intact PML NB or expression of the PML protein [63]. However, mechanistic explanations for these observations have not been provided as of yet.

### 2.5. The Role of Cytoskeleton during Late Events

PV utilize the cytoskeleton at late stages of the viral life cycle as well. Viral genomes are maintained in infected cells as extrachromosomal elements. Following establishment of infection with up to 100 copy numbers, viral genome amplification on average is restricted to approximately once per S phase resulting in a fairly constant copy number. During mitosis, viral minichromosomes attach to the cellular chromosomes via the E2 protein. Thus, they rely on the host cell machinery for partitioning of genomes during cell division ensuring that both daughter cells retain equal numbers of the viral genome. However, PV types have evolved divergent strategies for tethering their genome to cellular chromosomes. BPV1 tethers the genome in complex with the cellular bromodomain protein, Brd4, via the transactivating domain of E2 [64], whereas HPV8 associates with regions of ribosomal DNA repeats located on the short arm of acrocentric chromosomes using an interaction domain located in the hinge region of E2 [65]. These findings have recently been reviewed in detail [66].

The viral E6 and E7 oncoproteins also influence chromosomal segregation by inducing centrosome aberrations [67]. E6 protein has been shown to trigger centrosome accumulation [68]; E7 protein, on the other hand, can cause centrosome overduplication [67]. Since these processes are mainly observed in persistently infected and/or transformed cells aberrations may be due to uncontrolled expression of both oncoproteins and may not occur in the productive life cycle. However, it is possible that they also occur late in the viral life cycle and go unnoticed due to the terminal differentiation process. Recent reviews have addressed the processes underlying these observations in more detail [69–71].

Vegetative viral genome amplification is observed in fully differentiated cells of the upper spinous cell layer in cells that are usually incompetent for DNA replication. This is followed by expression of late proteins, including the E4 protein, and virion assembly. Since these terminally differentiating cells completely degrade their nuclei and viruses are released from the dying cells, cytoskeleton is not believed to play any role in the egress of progeny virions in contrast to many other viruses [72]. However, differentiated keratinocytes of the layer allowing late gene expression are highly keratinized. The dense keratin network has been proposed to hamper the release of progeny virus. A number of reports provide strong evidence that E4 protein, which is the most highly expressed viral protein, induces the depletion of keratin from these cells [73,74]. Keratin depletion is probably achieved by phosphorylation and ubiquitylation of differentiation-dependent and -independent keratins, which results in targeting of keratins for proteasomal degradation and keratin network disruption.

## 3. Conclusions

The development in recent years of novel experimental systems and tools has greatly enhanced our current understanding of the complex papillomaviral life cycle. However, many open questions remain. Hopefully, more systemic use of current technology and availability of improved and additional model systems in the future will allow answering the remaining questions to shed more light onto this fascinating group of human and animal pathogens.
